# Electroacoustic Analysis and Optimization of Needle-Rod Electrodes for Low-Power Impulse Sound Source

**DOI:** 10.3390/s25072331

**Published:** 2025-04-07

**Authors:** Xiao Du, Jing Zhou, Xu Gao

**Affiliations:** 1 College of Petroleum Engineering, Xi’an Shiyou University, Xi’an 710065, China; 22111010007@stumail.xsyu.edu.cn (X.D.); 20111010002@stumail.xsyu.edu.cn (X.G.); 2 National Engineering Laboratory for Oil and Gas Drilling Technology, Xi’an Shiyou University, Xi’an 710065, China

**Keywords:** impulse sound source, needle-rod electrodes, system power, sound pressure level, impulse wave intensity

## Abstract

In acoustic deep detection technology, conventional monopole, dipole, and phased-array sound sources are far inferior to impulsive sound sources in frequency and amplitude. But impulse sound sources mostly work under high-power, high-voltage, and high-current conditions, which are difficult to be applied downhole. The purpose of this paper is to reduce the power of the impulse sound source system and at the same time to stimulate excellent impulse wave characteristics. Firstly, an experimental impulse sound source system using needle-rod electrodes was constructed, and the discharge experimental results were analysed. Secondly, a finite element model of the needle-rod electrodes of the impulse sound source was established based on the experimental conditions, and the effects of the charging voltage, electrode gap, and liquid conductivity on the power and electroacoustic parameters of the needle-rod electrodes system were investigated separately. Finally, the optimised electroacoustic parameters and curves of the needle-rod electrodes of the low-power impulse sound source were obtained. The results show that the charging voltage is the most significant parameter affecting the power of the needle-rod electrode system; a larger liquid conductivity and a suitable electrode gap are required for the optimal impulse wave parameters. The optimised low-power impulse sound source system with needle-bar electrodes with a power of 20.95 kW achieves an impulse wave intensity of 4.78 MPa, with a sound pressure level above 295 dB up to 1 kHz and above 225 dB from 1 kHz to 300 kHz. Optimised needle-rod electrodes for low-power impulse sound sources have the advantages of a wide bandwidth and high energy. This makes the downhole application of low-power impulse sound sources possible, which will play an important role in oil exploration and other drilling exploration fields.

## 1. Introduction

Well logging is an important step in petroleum exploration, which is a method of measuring the physical parameters of the rock next to the well using geophysical properties, such as the electrical conductivity, electrochemistry, acoustics, and radioactivity of the rock formation in the well bore [1,2]. Acoustic logging is one of the many logging methods that is used to judge the quality of cemented wells [3] and evaluate the nature of rocks [4,5,6] by measuring the changes in speed, amplitude, and frequency of sound waves as they propagate through the medium. Traditional acoustic logging technology has a high detection accuracy, but the detection depth is shallow and it is difficult to observe trends in the extension and development of cracks next to the wells; seismic exploration has a larger detection depth, but the resolution is low and it is difficult to portray small geological bodies, such as fault blocks [7]. In recent years, drilling acoustic deep detection technology has been developing rapidly, which is superior to seismic exploration in terms of detection resolution, while the detection depth breaks through the limitations of traditional acoustic logging. The application of this technology is very promising. It can be used to display geological interfaces intersecting with wells; to detect inclined stratigraphic interfaces next to wells, cracks, faults, cusp extinctions, and the internal structures of salt mounds, etc.; to trace the oil storage boundaries in horizontal wells; and to detect stratigraphic information in front of the bit during drilling in order to decide on the next drilling direction and location for geosteering [8,9,10,11,12].

The sound sources of the acoustic depth sounding technology with drilling are classified as monopole sound sources, dipole sound sources, phased-array sound sources, and impulse sound sources. Research on monopole sound sources has been conducted [13,14]. The excitation frequency of monopole sound sources is generally around 10 kHz, the sound pressure level is around 132 dB, the emitted sound wave is greatly attenuated in the process of ground propagation, and the detection range is constrained to only a few metres to several dozen metres. The three-dimensional acoustic field excited by the monopole sound source is approximately spherical and does not have azimuthal directivity, so it cannot give specific information about the orientation of the reflector next to the well [15,16,17]. A dipole sound source consists of two point sound sources in close proximity to each other, which are equal in intensity and opposite in phase (180° difference). The excitation frequency of dipole sound sources is about 2–5 kHz, the sound pressure level is between 50 dB and 80 dB, the detection range is larger than that of monopole sound sources though the sound pressure level is low, the radiation directivity is in the shape of ‘∞’, and the orientation recognition has an uncertainty of 180°, which restricts the scope of their application [18,19,20,21,22]. A phased-array sound source consists of a plurality of vibrating elements, and the individual array elements may be distributed in a line, on a plane, or on a three-dimensional surface. The directional radiation of acoustic waves can be achieved by applying regular excitation signals (phase control and amplitude control) to each array element. Phased-array sound sources have the advantage of evaluating the spatial position of geological bodies next to wells and imaging three-dimensional formations, but the disadvantage is that the array combination is limited by the instrument space [23,24,25]. The excitation energy of the phased-array sound source is small, and the energy radiated to the outside of the well is limited, and the detection range is constrained.

Impulse sound sources are generated based on the hydroelectric effect. The obtained impulse wave is generated by a transient discharge in the electrode gap in water by high-voltage loading at both ends of the impulse capacitor. An impulse sound source has a wide bandwidth and high energy, can aggregate, is controllable, is capable of repeatable excitation, etc., which can better solve the problems of the existing sound source, and has a significant advantage in the application of acoustic wave deep detection [26,27]. An impulse sound source generally works under high-power, high-voltage, and high-current conditions [28], the system safety and stability requirements in practical applications are high, the discharge electrode ablation is serious [29], and the number of impulse capacitor discharges decreases. The impulse capacitors and control switches of an impulse sound source are large in size, which makes them difficult to install in the narrow space of the downhole detection instrument [30]. Therefore, further research is needed on how to reduce the system power of impulse sound sources and at the same time ensure they have excellent electroacoustic characteristics.

Therefore, in this paper, we design and build a impulse discharge experimental system for an impulse sound source needle-rod electrode in water. The effects of the charging voltage, electrode gap, and liquid conductivity on the power and electroacoustic parameters of the needle-rod electrode system of the impulse sound source are investigated by means of finite element modelling. The finite element model is used to study the influence of the charging voltage, electrode gap, and liquid conductivity on the power and electroacoustic parameters of the needle-rod electrode system of the hedge acoustic source. The optimum discharge parameters of the needle-rod electrode of the low-power impulse sound source are determined. Finally, the electroacoustic characteristic parameters and curves of the needle-rod electrode of a low-power impulse sound source are obtained. This paper lays the foundation for the application of needle-rod electrodes of low-power impulse sound sources in the field of drilling exploration.

## 2. Methods

### 2.1. Principle

The high voltage at both ends of an impulse capacitor acts on the electrodes in the liquid, generating a pulse discharge in a very short period of time, releasing the energy stored in the impulse capacitor instantaneously, and generating a huge impulse wave in the fluid medium, which is accompanied by strong radiation, a phenomenon known as the “hydroelectric effect” [31]. The hydroelectric effect is a high-speed conversion of energy, from electrical energy through discharge electrodes to other forms of energy such as sound, light, heat, and force.

**Figure 1:**
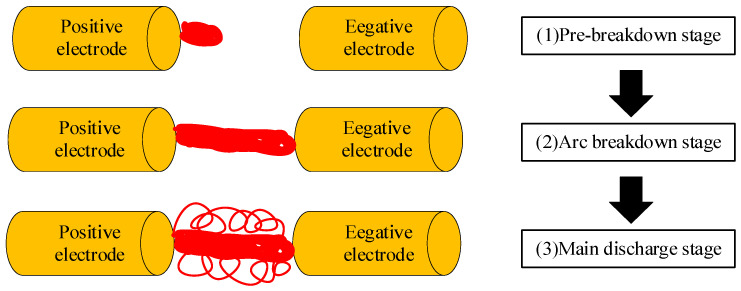
Process of impulse wave generation.

The process of impulse wave generation is divided into three stages. As shown in Figure 1, they are the pre-breakdown stage, the arc breakdown stage, and the main discharge stage.

The pre-breakdown stage is the process to generate the flow injection of the discharge electrode gap. In this paper, the model analysis of discharge in liquid of impulse sound source is mainly based on the bubble theory, which is combined with the electrostriction theory and direct ionisation theory to explain the phenomenon of liquid electric pulse discharge [32]. The discharge electrode gap is applied to strengthen the electric field, resulting in a field effect that generates tiny bumps on the electrode surface. Tiny bumps cause local electric field enhancement, and then micro-bubbles are formed due to the Joule heating effect of the surrounding liquid medium. Micro-bubbles and pre-existing bubbles in the liquid medium form an important basis for subsequent bubble breakdown theory.

The arc breakdown stage is a developmental extension of the initial arc (flow injection) to the other electrode. Under the continuous action of the applied electric field, if the boundary boiling temperature of the electrode water gap at this time T > 773.15 K and the average field strength of the gap E_b_ > 8 kV/cm, it is considered that the electrode breaks down and a plasma channel is formed.

When the plasma channel is considered as a cylindrical model, the time-varying resistance of the channel is as follows [33,34,35]:

(1) $$R_{ch}=\frac{d}{\sigma_{c}\pi t^{2}(t)},$$

(2) $$\sigma_{c}=1.411\times 10^{-2}T^{\frac{3}{2}}e^{\frac{5000}{T}},$$

where d is the channel length, and σ_c_ is the plasma channel conductivity.

**Figure 2:**
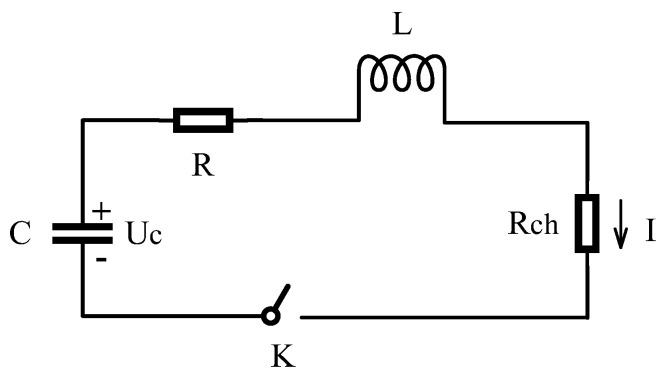
Discharge equivalent circuit.

The plasma channel time-varying resistance R_ch_ closely links the plasma channel motion process with the impulse sound source discharge circuit. The discharge equivalent circuit is shown in Figure 2, where Uc is the charging voltage of the energy storage capacitor, L and R are the equivalent inductance and equivalent resistance of the discharge circuit, R_ch_ is the time-varying resistance of the discharge plasma channel, and I is the current flowing through the plasma channel.

The main discharge stage is the process of rapid expansion of the plasma channel, resulting in a discharge current and a strong impulse sound wave. With the continuous support of high field strength, the energy in the pulsed capacitor is rapidly injected into the plasma channel and converted into channel deposition energy. Channel deposition of energy promotes the accelerated expansion of the arc and extrusion of the water medium outside the channel, due to the incompressibility of the water medium, producing impulse waves but also light, sound, and other radiation phenomena.

Defining the moment of plasma discharge breakdown as the initial moment, the initial conditions of the control equations are determined by the individual physical quantities of the equipartition channel state at the initial moment. The initial conditions of the plasma control equation are as follows:

(3) $$q_{0}=CU_{b},$$

(4) $$I_{0}=\frac{U_{b}}{R+R_{0}},$$

(5) $$n_{0}=\frac{P_{0}}{KT_{0}},$$

where U_b_ is the voltage at the time of discharge breakdown, R is the equivalent loop resistance, R_0_ is the initial resistance of the channel, P_0_ is the initial pressure of the channel, and T_0_ is the temperature of the channel at the breakdown time.

During the intense discharge, due to the Coulomb force and shrinkage effect between the particles, the modified gas equation of state is used to describe the pressure P in the plasma channel.

(6) $$P=nkT-\frac{\mu_{0}I^{2}}{8\pi^{2}{R_{ch}}^{2}}-\frac{e^{2}}{32\pi^{2}\varepsilon_{0}}{\left(\frac{4\pi n}{3}\right)}^{\frac{4}{3}},$$

where vacuum permeability μ_0_ = 4π × 10^−7^ H/m, vacuum dielectric constant ε_0_ = 1.6 × 10^−19^ F/m, unit charge e = 1.6 × 10^−19^ C, Boltzmann constant k = 1.38 × 10^−23^ J/K, plasma channel temperature is T, particle number density is n, and channel current is I.

The intensity of the impulse wave at the level D from the centre of the discharge electrode gap is as follows:

(7) $$P_{r}=\frac{P\cdot a}{D},$$

where a is the channel radius.

The Fourier transform of Equation (7) yields the impulse wave intensity in the frequency domain, P_rf_. Substituting P_rf_ into (8) yields the sound pressure level versus frequency.

(8) $$SPL=20\log_{10}\frac{P_{\mathrm{rf}}}{P_{\mathrm{ref}}},$$

where P_ref_ is the reference sound pressure, typically 1 × 10^−6^ Pa.

### 2.2. Experiment

**Figure 3:**
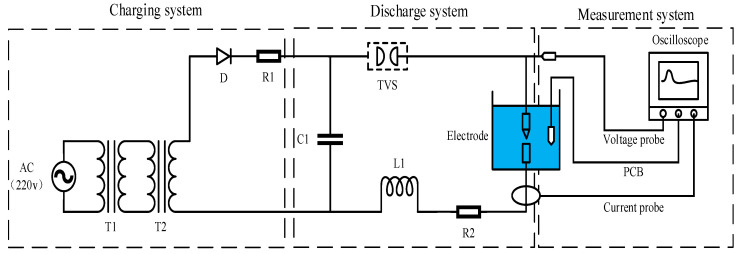
Schematic diagram of the experimental system of needle-rod electrode.

The principle of the experimental system of needle-rod electrode discharge for impulse sound source is shown in Figure 3. The experimental system consists of a charging system, a discharge system, and a measuring system. The charging system is powered by alternating current (220 V), which is boosted by a transformer and then rectified by silicon stack D. After rectification, the pulse capacitor C1 is charged. The discharge system waits for the capacitor to reach the charging voltage, then the triggered vacuum switch (TVS) conducts, the high voltage on capacitor C1 is instantly loaded onto the discharge electrode in the liquid, and the electrode gap is instantly broken. The measurement system uses a high-voltage probe (P6015A, Tektronix, Beaverton, OR, USA) to measure the electrode gap voltage, a current probe (Pearson 1330) to measure the electrode gap current, and a PCB underwater excitation pressure sensor (W138A01) at a level of 0.18 m from the centre of the electrode gap to measure the intensity of the impulse wave, and the measured data are synchronously recorded on an oscilloscope.

The structural parameters of the needle-rod electrode used in the experiment are shown in Table 1.

The external circuit and liquid environment parameters are shown in Table 2.

**Figure 4:**
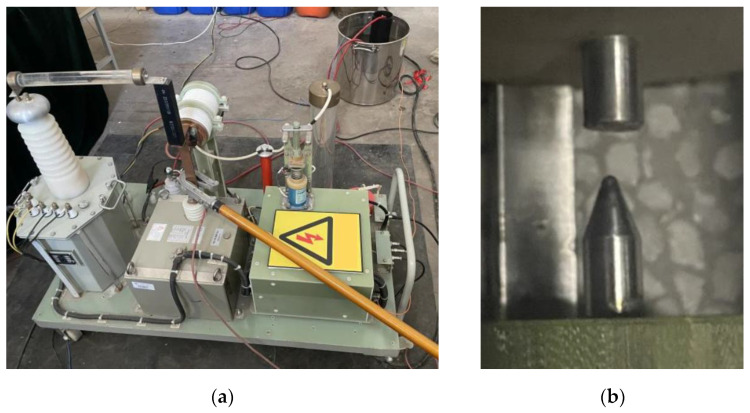
Discharge experiment system: (**a**) Overall diagram of the needle-rod electrode discharge experiment system; (**b**) Needle-rod electrode used in the experiment.

The impulse sound source experimental system using needle-rod electrode discharge is shown in Figure 4. Figure 4a shows an overview of the impulse sound source experimental system using needle-rod electrode discharge, and Figure 4b shows the needle-rod electrodes used in the experiment. The materials of the needle electrodes and rod electrodes are both stainless steel.

By triggering the control box to control the discharge of the pulse capacitor, the high voltage at both ends of the pulse capacitor is applied to both ends of the needle-rod electrode, which instantly breaks down and generates a strong impulse wave. The intensity of the impulse wave is related to the voltage value recorded by the pressure probe of the oscilloscope, which is converted according to Equation (9).

(9) $$P=\frac{U_{e}}{S}$$

where U_e_ is the effective value measured by the oscilloscope, S is the sensitivity of the pressure sensor, S = 0.73 mV/kPa, and P is the impulse wave intensity.

**Figure 5:**
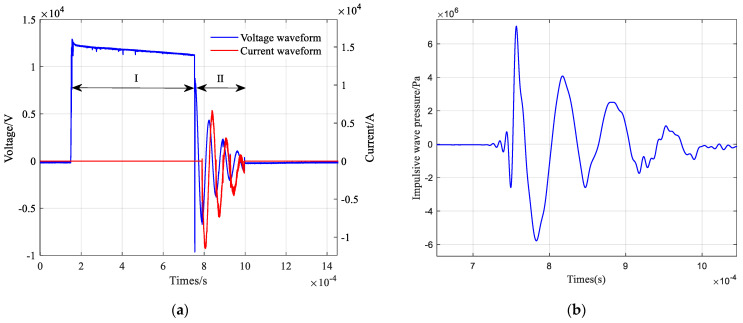
Experimental results of needle-rod electrode discharge: (**a**) Voltage and current curves; (**b**) Impulse wave intensity curve.

The experimental results of the needle-rod electrode discharge of the impulse sound source are obtained after conversion as shown in Figure 5. Figure 5a shows the voltage and current curves of the discharge experiment, and Figure 5b shows the intensity curve of the impulse wave. From the voltage and current curves, it can be seen that the curves show second-order oscillatory decay after the breakdown of the needle-bar electrode. Stage I (155.4~751.7 µs) is the pre-breakdown stage and arc breakdown stage, and the pre-breakdown time of the needle-rod electrode is calculated as 596.3 µs. Stage II (751.7~1000 µs) is a violent discharge process, and it can be seen from the voltage waveforms that the needle-rod electrode breaks down at the end of Stage I with a breakdown voltage of 11.26 kV. From the discharge current waveform, it can be seen that the peak value of the first wave of the current reaches 11.23 kA. From the impulse wave intensity waveform, it can be seen that the maximum value of the impulse wave intensity measured by the PCB underwater excitation wave pressure sensor is 6.95 MPa.

### 2.3. Validation

**Figure 6:**
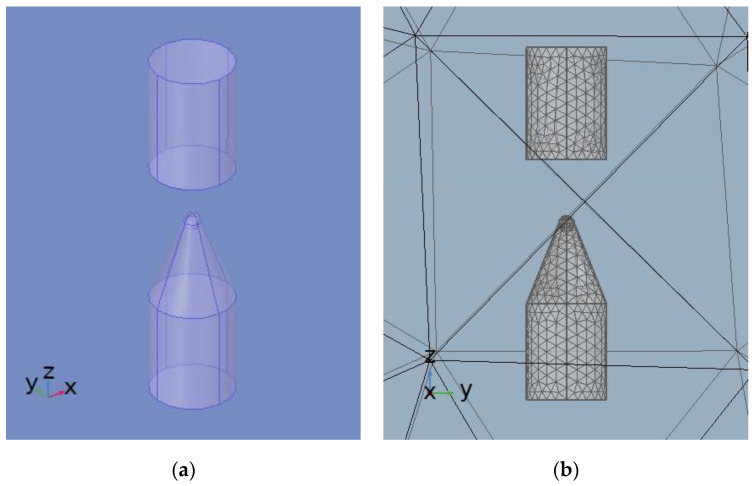
Finite element model: (**a**) Needle-rod electrode model; (**b**) Finite element meshing results.

The physical fields in the finite element model are selected as current field and fluid heat transfer field, and the multi-physics field is coupled with electromagnetic-thermal multi-physics field for transient simulation calculations [36,37]. According to the schematic diagram of the experimental system of impulse sound source using the needle-rod electrode, the finite element model of impulse sound source using the needle-rod electrode was constructed using finite element software as shown in Figure 6. Modelling was carried out in a 3D Cartesian coordinate system, needle-rod electrodes were constructed using the parameters in Table 1, and the external circuit and liquid environment were set up using the parameters in Table 2. The needle-rod electrode model is shown in Figure 6a, where the needle electrode is connected to the positive terminal of the output of the external circuit and the stick electrode is connected to the negative terminal of the output of the external circuit.

Figure 6b shows the results of mesh sectioning of the finite element model. Considering the fact that the discharge process is mainly concentrated in the tip of the needle-rod electrode and the medium near the tip, grid refinement was considered in the main discharge region of the electrode, and the grid density was assigned to different grid regions on demand. Numerical simulations at different grid densities can ensure accuracy while reducing the amount of computation and computation time.

In the discharge process, only the basic laws in the coupling of the current field and fluid heat transfer are followed, only the changes caused by heat conduction in the water and Joule heating of the current are taken into account, and the momentum generated by the water itself is not considered.

**Figure 7:**
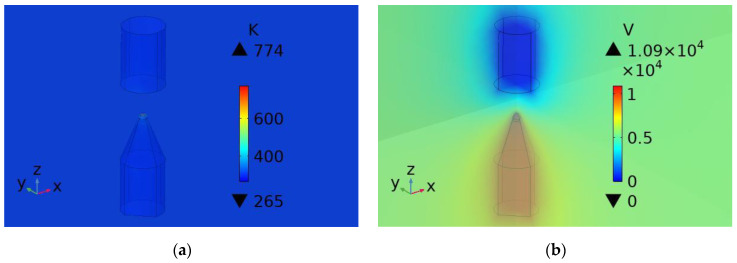
Breakdown time: (**a**) Temperature diagram; (**b**) Electric potential diagram.

The temperature and electric potential diagrams of the needle-rod electrode breakdown time of the impulse sound source are calculated and shown in Figure 7. Figure 7a is the temperature diagram at the breakdown time, and Figure 7b is the electric potential diagram at the breakdown time. At the moment of breakdown (602.56 μs), the temperature is 774 k, which is greater than 773.15 K, and the voltage at the moment of breakdown is 10.9 kV. It can be calculated that the average electric field strength of the electrode gap E_b_ is 15.57 kV/cm, which is greater than 8 kV/cm and that at the moment of needle-rod electrode breakdown.

The initial values obtained were calculated by simulation by substituting them into Equations (6) and (7), and the results obtained were compared with the experiments as shown in Table 3. It can be seen that the simulation results are more compatible with the experimental data, which verifies the correctness of the finite element model and lays the foundation for later calculating the influencing factors of the impulse wave electroacoustic parameters.

## 3. Discussion

The charging voltage, electrode gap, and liquid conductivity are important influencing factors on the power and electroacoustic parameters of the impulse sound source system using the needle-rod electrode, which directly affect the pre-breakdown time of the needle-rod electrode of the impulse sound source, the impulse wave intensity, the sound pressure level, and other parameters. The rest of the conditions of the model were set consistently when studying the law of influence of each parameter. Table 4 shows the parameter ranges and step sizes for each influencing factor studied in this paper.

### 3.1. System Power

**Figure 8:**
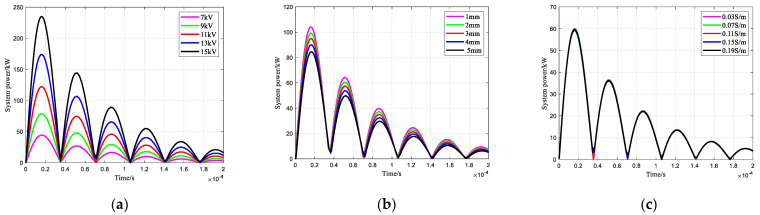
Changing law of system power: (**a**) Effect of charging voltage; (**b**) Effect of electrode gap; (**c**) Effect of liquid conductivity.

The multiplier of the discharge voltage and discharge current is the power consumed by the system. The lower the system power, the less energy the system requires, and the smaller the size of the pulse storage capacitor, which makes it easier to install downhole instruments in confined spaces and is beneficial to the system’s longevity, insulation safety, and stability. The patterns for the charging voltage, electrode gap, and liquid conductivity on the system power are shown in Figure 8. Figure 8a shows the effect of the charging voltage on the system power; it can be seen that the system power increases with increasing charging voltage. When the pulse capacitor charging voltage is 15 kV, the maximum value of the system power is 234.7 kW, and when the charging voltage is 7 kV, the maximum value of the system power is 44.37 kW, which shows that reducing the charging voltage can significantly reduce the system power.

Figure 8b shows the effect of the electrode gap on the system power, from which it can be seen that the system power decreases with the increase in the electrode gap, so increasing the electrode gap is also an effective way to reduce the power of the impulse sound source system with the needle-rod electrode. Figure 8c shows the effect of liquid conductivity on the system power, from which it can be seen that the liquid conductivity has almost no effect on the system power. Therefore, an effective way to reduce the system power is to reduce the charging voltage and increase the gap between the electrodes.

### 3.2. Pre-Breakdown Time and Gap Average Field Strength

**Figure 9:**
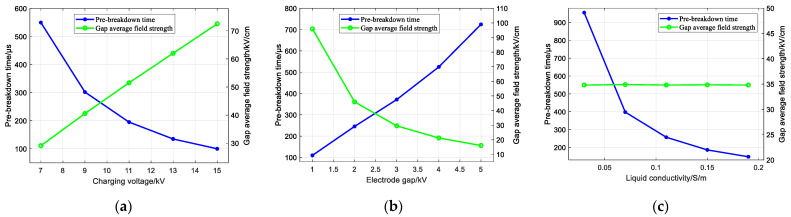
Changing law of pre-breakdown time and average electric field strength: (**a**) Effect of charging voltage; (**b**) Effect of electrode gap; (**c**) Effect of liquid conductivity.

The patterns of influence of the charging voltage, electrode gap, and liquid conductivity on the pre-breakdown time and gap average field strength of the needle-rod electrode of the impulse sound source are shown in Figure 9. Figure 9a shows the law of the effect of different charging voltages on the pre-breakdown time of the needle-rod electrode of the impulse sound source and the gap average field strength. From the figure, it can be seen that the pre-breakdown time gradually decreases and the gap average field strength gradually increases with the increase in charging voltage. Increasing the charging voltage is more favourable to the breakdown of the needle-rod electrode of the impulse sound source.

Figure 9b shows the influence laws of the pre-breakdown time and gap average field strength of the needle-rod electrode of the impulse sound source for different electrode gaps. From the figure, it can be seen that as the electrode gap increases, the pre-breakdown time becomes larger and the gap average electric field strength becomes smaller. Therefore, increasing the electrode gap distance is unfavourable for the breakdown of the needle-rod electrode of the impulsive sound source. Figure 9c investigates the influence of the pre-breakdown time and the gap average field strength of the needle-rod electrode of the impulse sound source for different liquid conductivities. From the figure, it can be seen that as the liquid conductivity increases, the pre-breakdown time is subsequently smaller, but increasing the liquid conductivity does not have an effect on the gap average field strength. Therefore, a larger liquid conductivity parameter should be selected.

### 3.3. Impulse Wave Intensity

**Figure 10:**
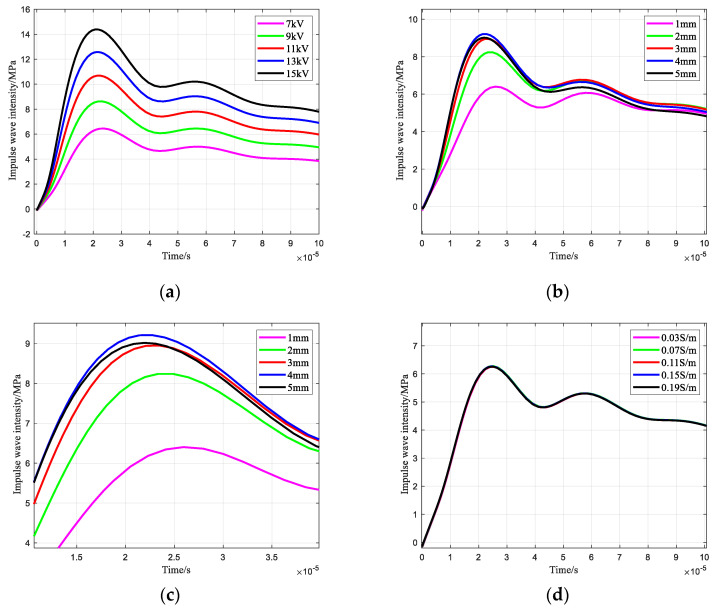
Changing law of impulse wave intensity: (**a**) Effect of charging voltage; (**b**) Effect of electrode gap; (**c**) Local amplification curve in Figure (**b**); (**d**) Effect of liquid conductivity.

The influence of the charging voltage, electrode gap, and liquid conductivity on the intensity of the impulse wave at the needle-rod electrode of the impulse sound source is shown in Figure 10. Figure 10a shows the effect of charging voltage on the impulse wave intensity. Figure 10b shows the effect of the electrode gap on the impulse wave intensity, and Figure 10c shows the local enlargement of Figure 10b, which shows that too small or too large of an electrode gap does not result in the maximum impulse wave intensity, and a suitable electrode gap (3 mm–4 mm) should be selected. Figure 10d shows the effect of liquid conductivity on the intensity of the impulse wave, and it can be seen that the liquid conductivity has almost no effect on the intensity of the impulse wave.

### 3.4. Sound Pressure Level–Frequency

**Figure 11:**
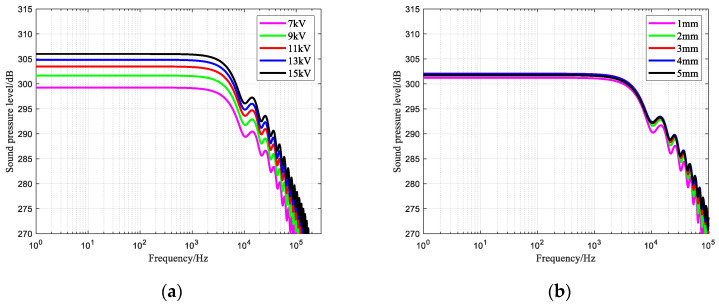

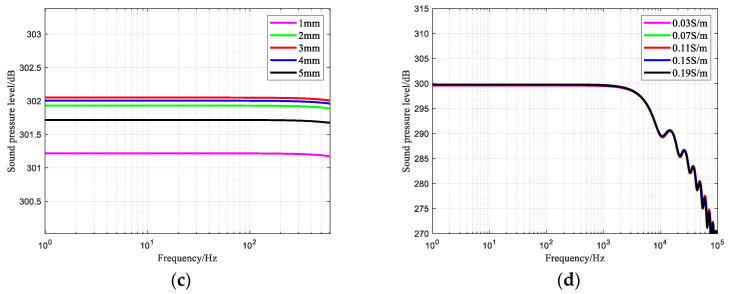
Changing law of sound pressure level–frequency of impulse sound source: (**a**) Effect of charging voltage; (**b**) Effect of electrode gap; (**c**) Local amplification curve in Figure (**b**); (**d**) Effect of liquid conductivity.

The variation rule of the sound pressure level–frequency of the needle-rod electrode of the impulse sound source under different charging voltages, electrode gaps, and liquid conductivities is shown in Figure 11. At the same frequency, the higher the charging voltage, the higher the sound pressure level. The smallest sound pressure level was found for a 1 mm electrode gap, followed by a 5 mm electrode gap. Therefore, too large or too small of an electrode gap is not necessarily advantageous for the optimisation of sound pressure level–frequency curves, and the selection of a suitable electrode gap is particularly important. The liquid conductivity has almost no effect on the impulse wave sound pressure level–frequency curve.

From the above analysis, it can be seen that the charging voltage has the greatest influence on the system power and electroacoustic characteristics of the impulse sound source, and although increasing the charging voltage can reduce the pre-breakdown time and can increase the gap average field strength, the impulse wave intensity, and the sound pressure level, it will not reduce the system power. The electrode gap needs to be chosen at the right spacing. For needle-rod electrodes, an optimum electrode gap of 3–4 mm will result in the best impulse wave intensity and sound pressure level–frequency curves. An increase in liquid conductivity significantly reduces the pre-breakdown time, so the liquid conductivity should be as large as possible.

## 4. Optimization

### 4.1. Optimization

In order to make the impulse sound source system have good electroacoustic characteristics while the power is reduced, a large liquid conductivity parameter and a suitable electrode gap (3–4 mm) are chosen as the excitation conditions for the optimised needle-rod electrode of the low-power impulse sound source. The choice of the charging voltage is a constraint for both reducing the system power and obtaining good electroacoustic parameters, and the charging voltage is the most significant factor influencing the system power. In order to reduce the system power, the smallest charging voltage at which the electrodes can break down should be used. Table 5 shows the process of determining the minimum charging voltage of the needle-rod electrode of the impulse sound source. The charging voltage is calculated starting from 10 kV and decreasing in steps of 1 kV, and after post-processing, the minimum charging voltage of the needle-rod electrode of the impulse sound source can be obtained as 6 kV.

The optimised electroacoustic parameters of the needle-rod electrode for the low-power impulse sound source are shown in Table 6, which were calculated for the needle-rod electrode of the impulse sound source under the conditions of a charging voltage of 6 kV, liquid conductivity of 0.19 S/m, and electrode gap clearance of 3.5 mm.

**Figure 12:**
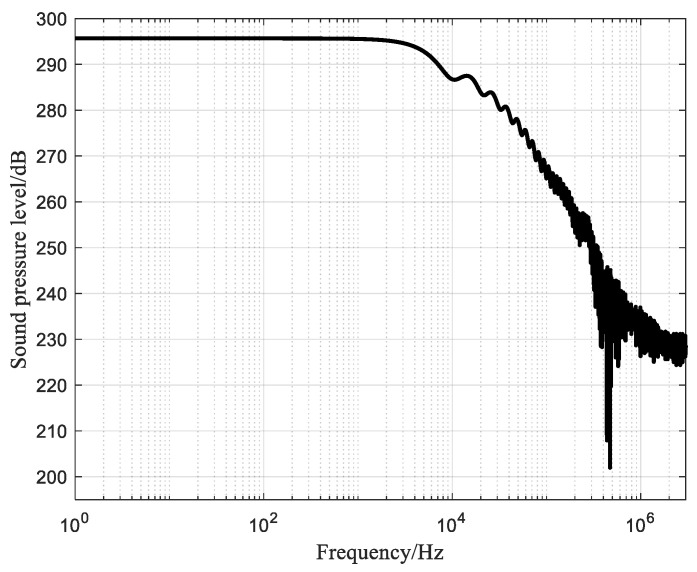
Sound pressure level–frequency plot of the needle-rod electrode of the low-power impulse sound source.

From Table 6, it can be obtained that the peak system power of the low-power needle-rod electrode is only 20.95 kW, and the impulse wave strength reaches 4.78 MPa. The optimised sound pressure level–frequency diagram for the needle-rod electrode of the low-power impulse sound source is shown in Figure 12. As can be seen from Figure 12, the sound pressure level is consistently maintained above 295 dB up to 1 kHz, which is advantageous for the detection of geological formations that are far away from the well. The 1–300 kHz sound pressure level starts to decay, but it is also above 225 dB, indicating that the needle-rod electrodes of the impulse sound source still have the characteristics of a wide source bandwidth and high sound pressure level, even though the power of the system is reduced.

### 4.2. Prospects

The impulse sound source has advantages in terms of the frequency band and sound pressure level, but research on low-power impulse sound sources is limited to theory and experiments. There may be the following problems in future engineering applications:

(1) Azimuthal characteristics: The sound field excited by the impulse sound source is a spherical wave, which does not have azimuthal radiation characteristics, so how to make the impulse sound source with azimuthal directivity is a key issue in the azimuthal detection of impulse sound sources;
(2) Adaptability to drilling: A downhole drilling environment with a high temperature, high pressure, and strong vibration poses challenges to the pulse energy storage capacitor and other components;
(3) Miniaturisation: The capacity of the storage capacitor and the volume of the storage capacitor are constrained by each other. For the narrow space of the downhole instrument, reducing the volume while ensuring the capacity of the storage capacitor is an issue that needs to be further considered for research.
(4) Data separation and processing of received signals: The drilling environment is complex, and the acoustic waves generated by the drill bit breaking up the rock and the vibration of the drill column will interfere with the echo signals. Meanwhile, a direct wave will also bury useful signals. It is worth studying how to separate useful echoes from complex signals.

## 5. Conclusions

In this paper, an impulse sound source experimental system using needle-rod electrode discharge is built, and the electroacoustic parameters of the needle-rod electrode are obtained by discharge. Then, through a numerical simulation, the influence laws of the charging voltage, electrode gap, and liquid conductivity on the system power and electroacoustic characteristics were analysed. The parameters and curves of the electroacoustic characteristics of the needle-rod electrodes under low-power operating conditions were finally obtained. The main conclusions are as follows:

(1) The impulse sound source works under high-power, high-voltage, and high-current working conditions, the level of electrode erosion is serious, the system insulation is poor, and the pulse capacitors, control switches, and other components are large; thus, the instrument is difficult to install in a small space;
(2) Reducing the charging voltage is the most significant way to reduce the power of the needle-rod electrode system for the impulse sound source, but it results in a weaker impulse wave intensity and sound pressure level. The optimum impulse wave can only be stimulated by a suitable electrode gap. A larger liquid conductivity reduces the pre-breakdown time of the needle-rod electrode of an impulse sound source;
(3) The optimised needle-rod electrode of a low-power impulse sound source system has a peak power of only 20.95 kW, a maximum impulse wave intensity of 4.78 MPa, and a sound pressure level of more than 295 dB below 1 kHz and more than 225 dB from 1 kHz to 300 kHz, which is characterized by a wide bandwidth and high energy.

## Figures and Tables

**Table 1:** Structural parameters of needle-rod electrode.

Structure	Size (mm)
Needle-cone top radius	1
Needle-cone bottom radius	5
Needle-cone height	10
Needle-column height	12
Rod radius	5
Rod height	14

**Table 2:** External circuit and liquid environmental parameters.

External Circuit Parameters		Size (mm)	
Charging voltage (kV)	12.8	Conductivity (S/m)	0.07
Energy storage capacitors (µF)	15	Initial temperature (K)	293.15
Equivalent resistance (Ω)	0.22	Hydrostatic pressure (Pa)	101,325
Equivalent inductance (µH)	8.18	Relative permittivity	81

**Table 3:** Comparison results between simulation and experiment.

Compared Parameters	Breakdown Voltage/kV	Pre-Breakdown Time/μs	Peak Breakdown Current/kA	Impulse Wave Intensity/Mpa
Experiment	11.26	596.3	11.23	6.95
Simulation	10.9	602.56	11.01	6.75
Percentage error	3.2%	1.05%	11.01	2.88%

**Table 4:** Influencing factors studied in this paper.

Factor	Range	Step Size
Charging voltage (kV)	7–15	2
Electrode gap (mm)	1–5	1
Liquid conductivity (S/m)	0.03–0.19	0.04

**Table 5:** Determination process of minimum charging voltage.

Charging Voltage (kV)	Breakdown Temperature (K)	Breakdown Voltage (kV)	Gap Average Field Strength (kV/cm)	Breakdown?
10	777	8.65	24.71	Yes
9	774	7.45	21.29	Yes
8	775	6.25	17.8	Yes
7	773	4.88	13.9	Yes
6	776	3.43	9.8	Yes
5	719	1.09	3.11	No

**Table 6:** Electroacoustic parameters of needle-rod electrodes for low-power impulse sound source.

Pre-Breakdown Time (µs)	Breakdown Voltage (kV)	Gap Average Field Strength (kV/cm)	Peak Breakdown Current (kA)	Peak Impulse Wave Intensity (MPa)	Peak System Power (kW)
606.9	3.43	9.8	3.53	4.78	20.95

## Data Availability

The original contributions presented in the study are included in the article; further inquiries can be directed to the corresponding author.
